# WEBINO: A unique neuro-ophthalmological manifestation of the unilateral mid-brain cerebrovascular event

**DOI:** 10.1016/j.radcr.2024.10.135

**Published:** 2024-11-30

**Authors:** Avinash Parepalli, Saket S Toshniwal, Jiwan Kinkar S, Sourya Acharya, Nikhil Reddy B

**Affiliations:** a Department of General Medicine, Jawaharlal Nehru Medical College, Datta Meghe Institute of Higher Education and Research, Wardha, India; b Department of Neurology, Jawaharlal Nehru Medical College, Datta Meghe Institute of Higher Education and Research, Wardha, India

**Keywords:** WEBINO, MLF, Mibrain, Cerebrovascular event, Abduction, Adduction

## Abstract

WEBINO (wall eye intranuclear ophthalmoplegia) is a specific type of neuroophthalmological condition that occurs due to a lesion in the MLF (medial longitudinal fasciculus), which causes unique symptoms characterized by bilateral adduction (inward movement of the eyes) impairment. Still, the abduction (outward movement of the eyes) may be preserved, and nystagmus during abduction is seen along with severe exotropia (outward movement of the eyes). In this case report, we report a 65-year-old male with a sudden onset of double vision, which he noticed while watching television, associated with a certain degree of vision loss and right-sided weakness of the body. Upon investigation, his imaging revealed an ischemic lesion in the unilateral midbrain involving his MLF. Lessons learned from this case are: This case spotlights the need for recognizing the WEBINO in cases of cerebrovascular events. It points out that for early diagnosis and proper treatment of WEBINO to achieve better results, there is a low incidence of stroke as the cause, more research is required, or better prognosis and treatment strategies in patients of WEBINO. It also underlines the need of comprehensive treatment measures, such as risk factor reduction and rehabilitation, for improving recovery and preventing future cerebrovascular episodes.

## Introduction

WEBINO, a bilateral kind of internuclear ophthalmoplegia, is less common than unilateral variations, affecting 1%-2% of those who have vertebrobasilar strokes and 2%-5% of ischemic stroke patients. However, accurate data on its frequency and prevalence are limited [1,2].

WEBINO is one of the very rare neuro-ophthalmological conditions characterized by lesions in the bilateral medial longitudinal fasciculus. The symptoms comprise major gaze exotropia, limited adduction, and nystagmus during abduction [3]. The most common causes are brain stem infarctions and multiple sclerosis; however, infections, inflammatory causes, trauma, and metabolic disorders are all conceivable. Depending upon the localization of the lesions in the brainstem, there might be variations in the presentation of WEBINO. The occurrence of WEBINO is rare in cerebrovascular events, and its occurrence in unilateral stroke makes it more unique than in our case. Internuclear ophthalmoparesis (IN) is a similar condition producing reduced horizontal eye movements and abnormal adduction and abduction nystagmus [4]. It is commonly precipitated by damage to the MLF from a pontine infarction or midbrain lesions that affect MLF fibers. Isolated unilateral INO (intranuclear ophthalmoplegia) is very uncommon and may arise from a small ischemic stroke to the MLF. The blood supply to the midbrain can result in certain infarction patterns [3,5].

WEBINO is one of the variants of INO with somewhat similar underlying etiologies. Few papers discuss prognosis and therapy, whereas genesis and pathophysiology often dominate the literature describing these conditions. Common presenting features include bilateral adduction deficiencies, abducting nystagmus, and large-angle exotropia. Multiple sclerosis is a common cause of internuclear ophthalmoplegia. Although research has led to a better understanding of the pathophysiology and causes of WEBINO, treatment techniques still need to be studied [3,5].

This report will outline the neurological circuits implicated in WEBINO, examine pathogenesis, and provide current insights on prognosis and therapy.

### Case

A 65-year-old man presented to the emergency room with an abrupt onset of double vision and sudden weakness on the right side of the body with difficulty in coordination. He reported that the symptoms have intensified during the last 12 hours. His ``weakness'' of reclining back or gazing to the side was his most difficult, although he experienced significant instability in his legs and mild impairments in his arms. He had felt moderately dizzy with a little imbalance when walking. He denied having a headache, nausea, vomiting, speech difficulty, facial drooping, or experiencing any recent head trauma. No recent infections or systemic diseases were noted.

**Fig. 1:**
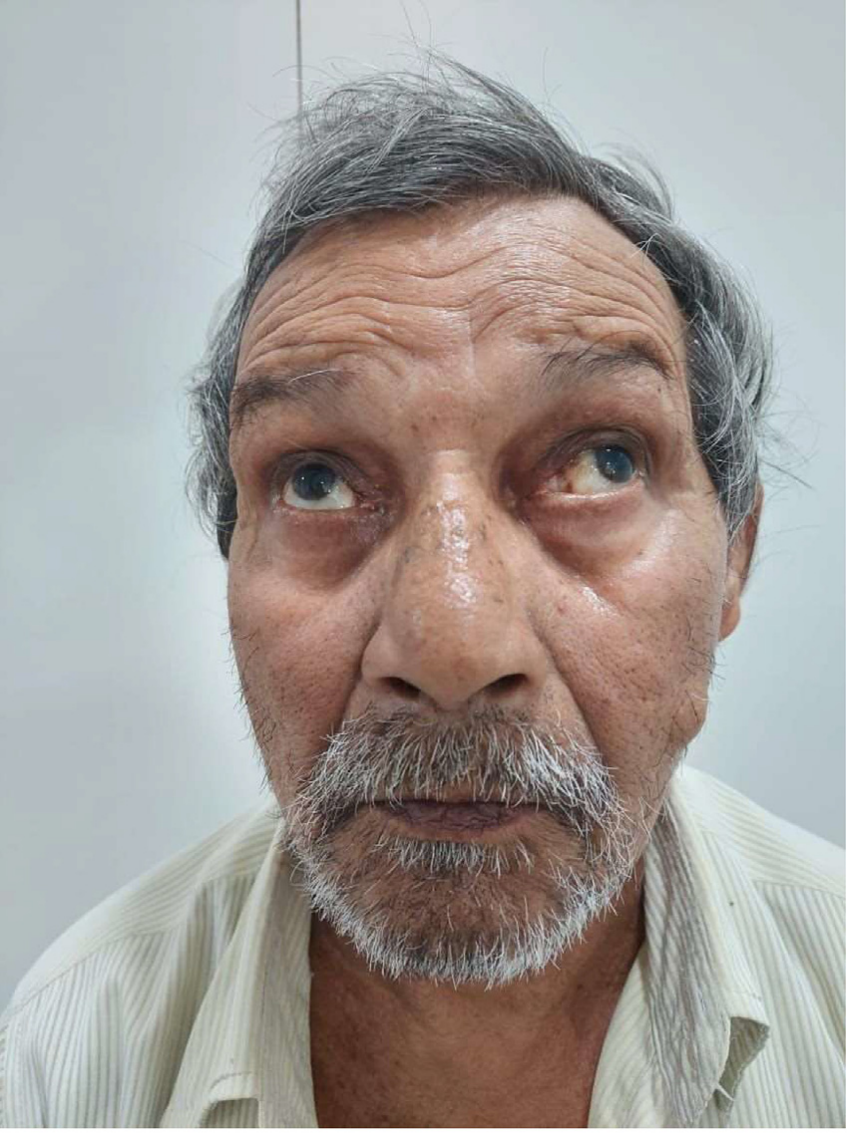
Shows the patient with bilateral abduction of the eyes.

The patient has been known to be hypertensive and hyperlipidemic for many years on regular medication and followup. He is on oral medication with cilnidipine 5 mg and rosuvastatin 20 mg daily. He is a chronic smoker with a 40-pack-year smoking history and a chronic alcoholic; he consumes 180-240 ml of alcohol daily. The patient's father had a history of stroke at 75 years of age. His sleep and appetite were normal,

The patient was conscious, coherent, and well oriented to time, place, and person. Upon examination, his blood pressure was 150/90 millimeters of mercury, his pulse was 88 beats per minute, and saturation at room air was 98%. The neurological evaluation comprised testing for cranial nerves, motor strength, sensation, coordination, and gait. A crucial discovery was in his eye movements: when asked to look to the left or right, the patient demonstrated bilateral weakness by turning both eyes inward toward the nose with abducting nystagmus as shown in Fig. 1, which refers to involuntary, jerky eye movements on the opposite side. His horizontal eye motions were aberrant, but his upward and downward gaze were normal. His pupils were equal and round and reactive to light; ptosis was absent. With this specific combination of eye movement abnormalities—adduction failure bilaterally with abducting nystagmus.

The motor assessment indicates significant right hemiparesis. The patient's right arm has a muscle strength of 2/5 and cannot lift or grasp anything. Similarly, a patient with right hemiparesis will be unable to stand or walk since the right leg has just 3/5 of its strength. Notably, the patient exhibits indications of incoordination in his right arm, with dysmetria seen when attempting to point his finger toward his nose and regularly failing to achieve the objective. During quick alternating movement tasks, the right arm moves in a clunky, unsteady manner indicative of ataxia. Furthermore, the neurological examination shows minor lower face paralysis on the right side due to drooping of the angle of the lips, and the forehead seems intact, indicating that it is an upper motor neuron. Eye movement has decreased, and the patient has previously complained of double vision, which might be an indication of oculomotor nerve dysfunction. Reflexes were evaluated, and the right side showed quick deep tendon reflexes as well as a positive babinski sign.

**Fig. 2:**
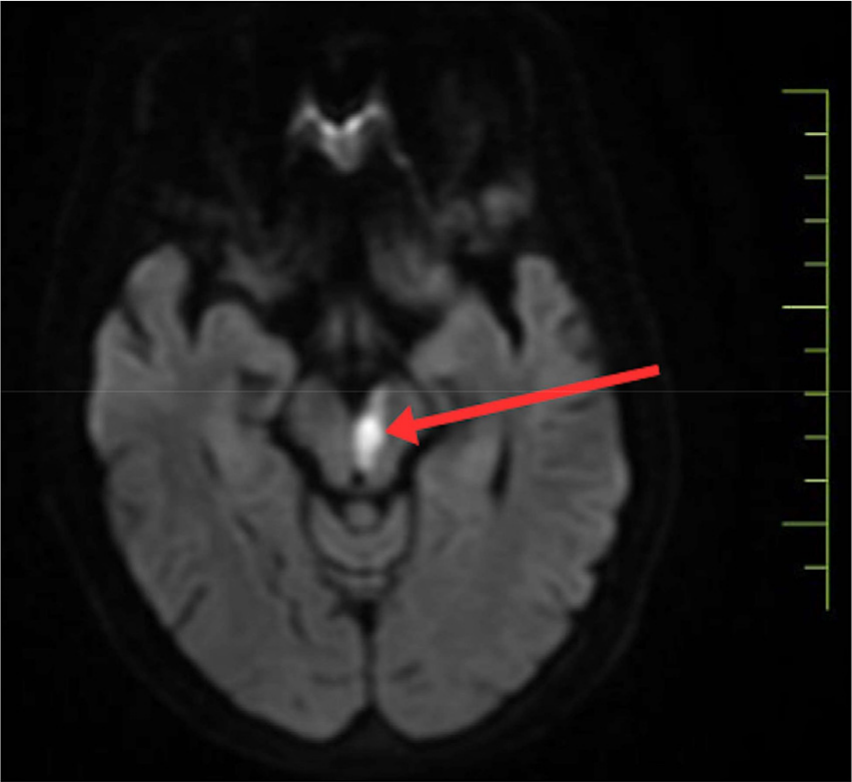
Shows the left paramedian midbrain's hyperintense area on diffusion-weighted imaging on MRI axial sections, indicating an acute infarct.

**Fig. 3:**
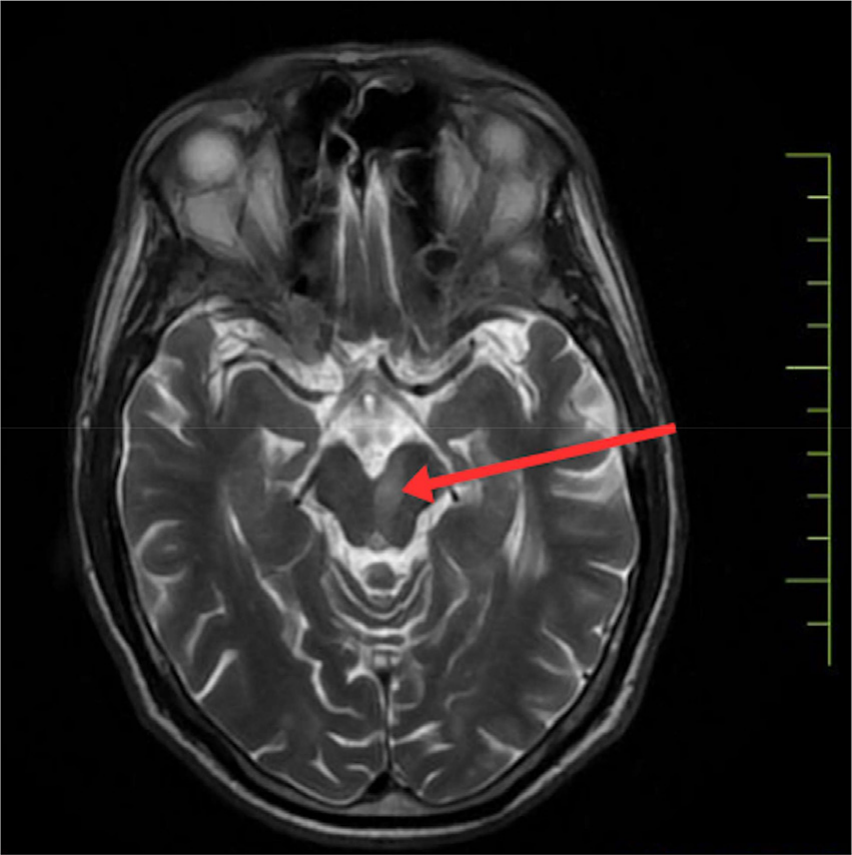
Shows the hyperintensity in the left paramedian midbrain on the patient's axial T2 section of the MRI.

**Fig. 4:**
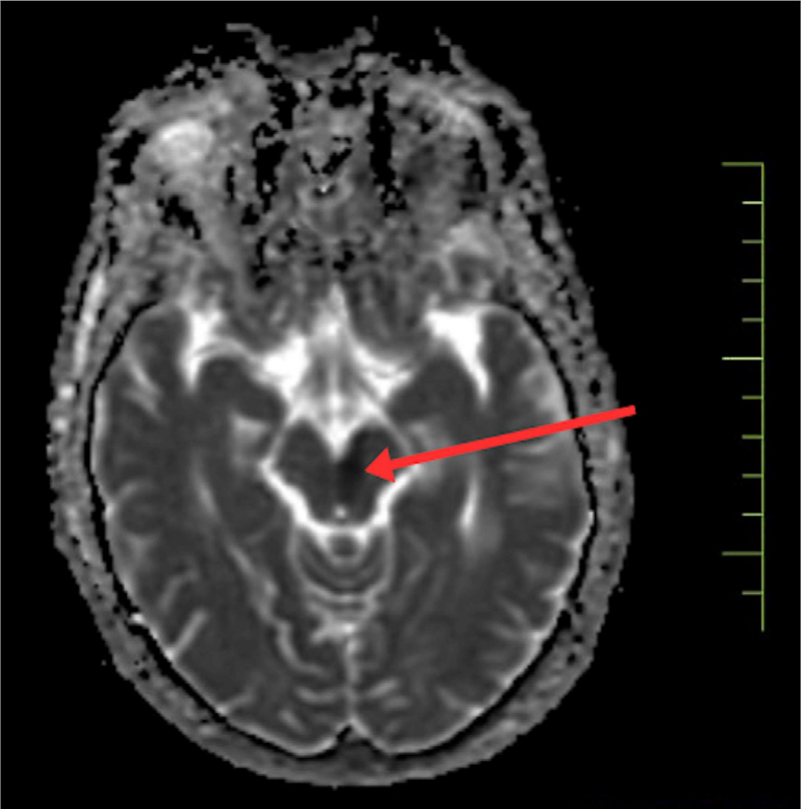
Shows the left paramedian midbrain's low intensity on the ADC view.

**Fig. 5:**
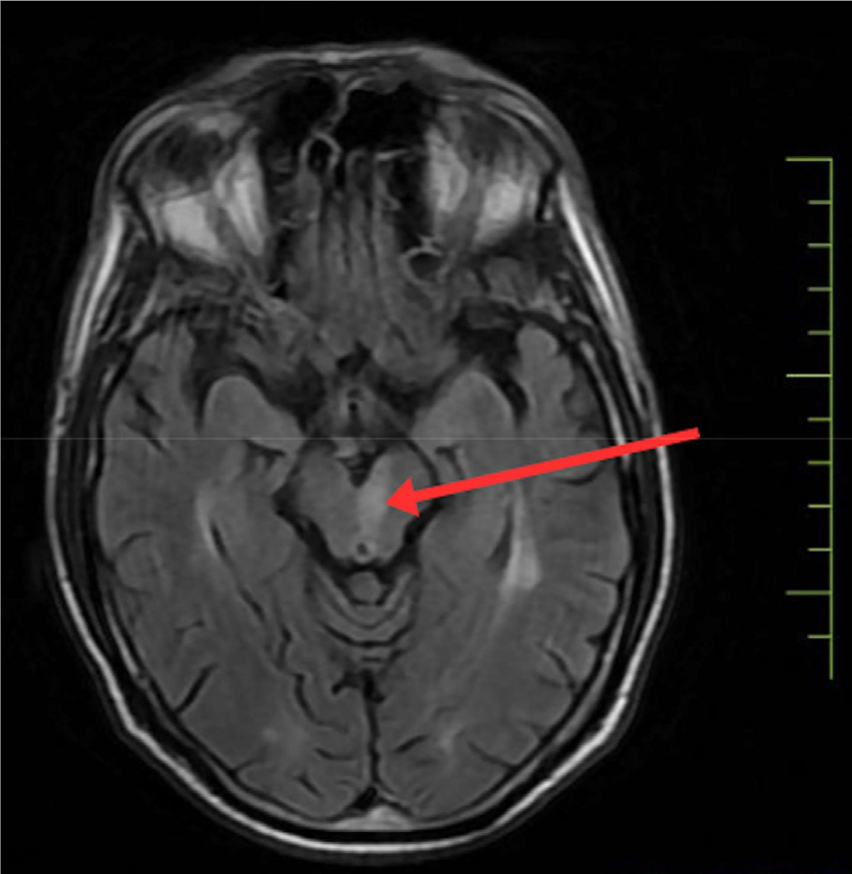
Shows the hyperintensity on the left paramedian midbrain in the axial section of the FLAIR view on MRI.

To confirm the diagnosis, urgent magnetic resonance imaging of the brain was done, which revealed, There is altered signal intensity noted in the left midbrain, which appears hypointense on T1, hyperintense on T2/FLAIR, shows restriction on DWI, low signal on ADC-suggestive of acute infarct. Areas of altered signal intensity are noted in the bilateral gangliocapsular and subcortical regions of the frontal lobe, which appear hypointense on T1 and FLAIR, hyperintense on T2WI; however, no restricted diffusion is noted s/o chronic lacunar infarcts. Prominence of sulcogyral spaces, sylvian cistern, and dilatation of the ventricular system—suggestive of age-related atrophic changes. Multiple discrete T2W/FLAIR hyperintensities noted in bilateral periventricular and subcortical white matter are suggestive of chronic small vessel ischemic changes. (FAZEKA'S GRADE III). The MRI imaging sections are shown in Fig. 2, Fig. 3, Fig. 4, Fig. 5

**Fig. 6:**
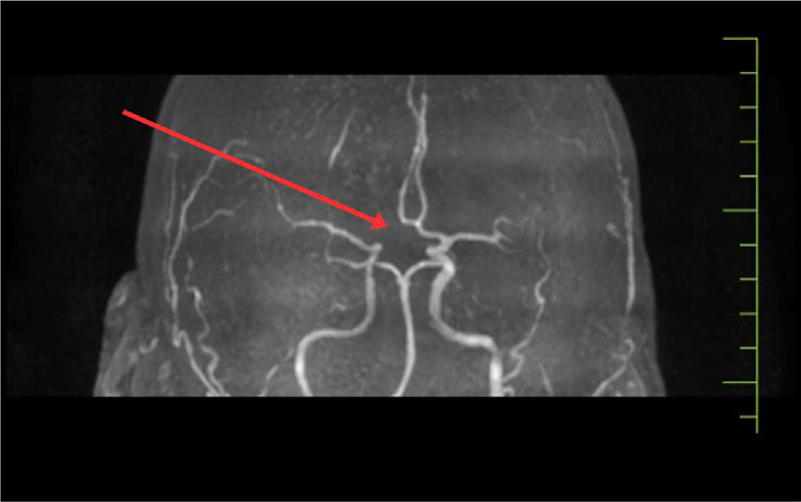
Showing the MR angiography of intracranial vessels, reveals the A1 segment of the left A.C.O.M. is hypoplastic and has a normal course.

MR angiography of intracranial vessels reveals the A1 segment of the left ACOM is hypoplastic and has a normal course, as shown in Fig. 6.

An additional cardiovascular workup was performed, an ecg revealed no arrhythmias other than atrial fibrillation, and transthoracic echocardiography revealed no signs of heart thrombosis or other structural abnormalities with good left ventricular systolic and diastolic function, EF 60%,

The patient presented 12 hours after symptom start, which was outside the time window for intravenous thrombolysis (tpa). He was thus treated cautiously, although with antiplatelet medication. A loading dosage of aspirin (300 mg), clopidogrel (300 mg), and injectable low molecular weight heparin of 0.6 ml subcutaneously was given. He was continued with a daily dose of aspirin (75 mg), clopidogrel (75 mg), low molecular weight heparin of 0.4 ml subcutaneous, and a high dose of statins (rosuvastatin 40 mg) for 7 days. During this time, extensive rehabilitation therapy (physiotherapy and speech therapy) was provided. His blood pressure was maintained with tab labetalol below 140/90 mm hg.

He was examined after 1 week and had improved slightly in terms of eye movement irregularities, but still showed diplopia when turning his eyes to either side. He had also improved his balance via physical treatment, and he was now working with his physiotherapist on some coordination exercises. He was placed on a regular follow-up schedule in the stroke clinic for continued evaluation of his level of recovery as well as risk factor treatment to avoid recurrence.

## Discussion

Stroke is now becoming a global health issue. In many developing countries, it has already become the number one cause of morbidity and mortality. Stroke is increasingly faced in India today and ranks fourth among the causes of mortality and fifth among the causes of morbidity. Previous studies suggested that the prevalence of stroke in India is between 105 to 152/100,000 persons per year [6,7].

In a study of 39 case reports comprising 42 WEBINO patients aged 12 to 85 years revealed that they had a variety of neuro-ophthalmological symptoms. The main eye position revealed exotropia (88.1%), bilateral intraocular disorders (90.5%), convergence barrier (52.4%), blepharoptosis (14.3%), vertical gaze disorders (42.9%), vertical nystagmus (14.3%), pupillary abnormalities (19.0%), and reversed dip (4.8%). The lesion locations were discovered to be in the midbrain (66.7%), pons (33.3%), both midbrain and pons (21.4%), and brainstem (2.4%). Morbidity was caused by cardiovascular and cerebrovascular disorders (61.9%), neurodiseases (23.8%), infectious diseases (7.1%), tumor-related diseases (4.8%), alcohol overdose (2.4%), and drug-immune responses (2.4%) [8]. WEBINO presentation is a rare inidence in cerebrovascular event and its occurrence in unilateral midbrain stroke as in our case is very rare.

WEBINO is a neurological condition that affects the performance of both horizontal conjugate eye movements and vestibular ocular movements. The 2 abducens nerve nuclei and the para-median pontine reticular formation (PPRF) are located in the poins from where the path way commences [3,9]. The superior colliculus and upper cortical areas send signals to the PPRF that further carries the signal to the ipsilateral abducens nerve nucleus thus there is a horizontal saccade. In the middle of the mid brain, is a pair of white matter tracts named MLF, which contains multiple fibers linking all ocular motor cranial nerve nuclei and also transferring vestibular information from the vestibular organs of the inner ear. WEBINO also has an effect on the vestibular eye movement pathways via abnormalities in the medial longitudinal fasciculus [4,10].

Due to the horizontal and vertical movements of the head, the semicircular canals stimulate the vestibulocochlear nerve, which provokes vestibulo-ocular refluxes. Excitatory impulses from the semicircular canals are transmitted to the ipsilateral vestibulocochlear nucleus and then through the medial longitudinal fasciculus to the contralateral abducens nucleus or contralateral oculomotor nucleus in the brainstem. The oculomotor and abducens nuclei are those that control horizontal and vertical eye movements, innervating several extraocular muscles and completing signal transduction [11].

Various theories have been proposed, but most of these have lacked valid evidence. Some scientists felt that webino-induced exotropia is due to a lesion in the pontine area of the brainstem or due to direct involvement of the nucleus of the oculomotor nerve in the midbrain. Further neuroimaging studies with a higher number of webino patients are needed to garner dependable findings on the etiology of exotropia that can classify webino as a separate type of internuclear ophthalmoplegia [4,10].

Abducting nystagmus is due to Hering's law of equal innervation. According to this, the yoked extraocular muscles receive an equal amount of innervation. Since the weaker medial rectus increases innervation to the medial rectus, there is greater innervation to the contralateral lateral rectus, resulting in nystagmus. Patients with Webino have vertical gaze problems that comprise reduced vertical gaze holding, nystagmus, saccades, pursuits, and vestibulo-ocular reflexes [5,9,11].

This is an example of bilateral internuclear ophthalmoplegia, which demonstrates the need to be vigilant and recognize the clinical indications of such a disorder, particularly in the setting of an acute ischemic stroke. As a result, rapid identification and implementation of appropriate stroke therapy techniques, including long-term prevention, are crucial for improving patient outcomes and preventing future cerebrovascular episodes.

## Conclusion

This report emphasizes the necessity of doctors having a high index of suspicion for webino in patients presenting with acute ocular motor difficulties, particularly when there are symptoms of cerebrovascular risk factors. Furthermore, it emphasizes the urgent necessity for ongoing study into the prognosis and treatment strategies for webino and other similar internuclear ophthalmoplegias to improve patient care and results in future cases. Frequent follow-up and supporting measures are used to promote maximum recovery and avoid recurring cerebrovascular episodes, therefore proving the significance of multidisciplinary care in rehabilitation for patients with complicated neurological presentations.

## Patient consent

The patient granted written consent to publish his clinical and MRI images. All obligations were, thus, met according to ethical standards and institutional regulations to respect the patient's rights and privacy. At this moment, the photographs are released for knowledge and education in medical practice but without the patient's identity. Information may be, therefore, removed to protect confidentiality.
